# FnR: R package for computing inbreeding and numerator relationship coefficients

**DOI:** 10.1186/s12862-024-02285-4

**Published:** 2024-07-18

**Authors:** Mohammad Ali Nilforooshan

**Affiliations:** Livestock Improvement Corporation, Private Bag 3016, Hamilton, 3240 New Zealand

**Keywords:** Breeding, Genetic diversity, Inbreeding, Numerator relationship matrix, Pedigree, R package

## Abstract

**Background:**

Inbreeding and relationship coefficients are essential for conservation and breeding programs. Whether dealing with a small conserved population or a large commercial population, monitoring the inbreeding rate and designing mating plans that minimize the inbreeding rate and maximize the effective population size is important. Free, open-source, and efficient software may greatly contribute to conservation and breeding programs and help students and researchers. Efficient methods exist for calculating inbreeding coefficients. Therefore, an efficient way of calculating the numerator relationship coefficients is via the inbreeding coefficients. i.e., the relationship coefficient between parents is twice the inbreeding coefficient of their progeny. A dummy progeny is introduced where no progeny exists for a pair of individuals. Calculating inbreeding coefficients is very fast, and finding whether a pair of individuals has a progeny and picking one from multiple progenies is computationally more demanding. Therefore, the R package introduces a dummy progeny for any pair of individuals whose relationship coefficient is of interest, whether they have a progeny or not.

**Results:**

Runtime and peak memory usage were benchmarked for calculating relationship coefficients between two sets of 250 and 800 animals (200,000 dummy progenies) from a pedigree of 2,721,252 animals. The program performed efficiently (200,000 relationship coefficients, which involved calculating 2,721,252 + 200,000 inbreeding coefficients) within 3:45 (mm:ss). Providing the inbreeding coefficients (for real animals), the runtime was reduced to 1:08. Furthermore, providing the diagonal elements of **D** in $\textbf{A} = \textbf{TDT}^{\prime }$ (**d**), the runtime was reduced to 54s. All the analyses were performed on a machine with a total memory size of 1 GB.

**Conclusions:**

The R package FnR is free and open-source software with implications in conservation and breeding programs. It proved to be time and memory efficient for large populations and many dummy progenies. Calculation of inbreeding coefficients can be resumed for new animals in the pedigree. Thus, saving the latest inbreeding coefficient estimates is recommended. Calculation of **d** coefficients (from scratch) was very fast, and there was limited value in storing those for future use.

## Background

An important point in any conservation program, or generally in any breeding program, is maintaining genetic diversity and monitoring and minimizing inbreeding. There are negative consequences of increased inbreeding in a population. Coincided with increased homozygosity, inbreeding increases the frequency of individuals expressing deleterious allele effects, which were present in the population at lower frequencies. Inbreeding depression is the reduced survival, fertility, and biological fitness of related individuals’ offspring [1]. This is more likely to happen in isolated and small populations. However, it may also happen in large populations such as in Holstein dairy populations, where a small group of males are ancestor to many progenies, reducing the effective population size. Effective population size and inbreeding are interconnected. Several methods exist for estimating effective population size (*Ne*) based on inbreeding rate ($\Delta F$, change of the average inbreeding coefficient over time). Those methods differ in how $\Delta F$ is derived but are similar in the relation $Ne = 1/2 \Delta F$ [2].

Inbreeding and its negative consequences (inbreeding depression) can be minimised in conservation or breeding programs by designing a mating plan that results in the lowest possible inbreeding rate in the next generation. Where possible, artificial insemination techniques are useful in ensuring that the mating plan proceeds as planned and that the mating is done at a proper time (Estrus or heat of the female animal) to increase the conception rate and the number of progeny. The increased number of progeny from more parent combinations will bring more opportunities to reduce inbreeding and increase genetic variation in the population.

Inbreeding directly reduces genetic variation by reducing heterozygosity and indirectly via inbreeding depression, reducing the number of parents to the next generations and the population size. The reduction in effective population size caused by inbreeding can lead to a population-scale reduction in genetic diversity as it amplifies genetic drift, which might put the population at risk of a genetic bottleneck. Genetic diversity is vital for livestock selection and genetic gain in any breeding program. It allows sustainable genetic improvement and facilitates adaptation to changing environments and breeding objectives [3].

Another parameter of interest is the additive genetic relationship coefficient between individuals. The additive genetic relationship between two individuals is twice their coancestry, and the coancestry or the coefficient of kinship is the probability of identical by descend genes passing off to the two individuals [4]. Additive genetic relationship coefficients among individuals are collated in a matrix called the numerator relationship matrix (**A**) using the pedigree information or the genomic relationship matrix (**G**) using genotype markers. The dimensions of these matrices are the number of individuals in the pedigree and the number of genotyped individuals, respectively. The diagonal element of **A** for animal *X* is twice the probability that two randomly chosen gametes from animal *X* will carry identical by descend alleles, and equals 1 + $F_X$, where $F_X$ is the inbreeding coefficient of individual *X* [5, 6].

With the availability of dense marker data, genomics plays an important role in monitoring genetic diversity and genomic selection in both small endangered and large commercial populations. However, pedigree information matters because

1. Currently, genotyped animals comprise a small proportion of the population in most species. For example, 1,424,863 of 20,367,132 cows registered between 2000 and 2021 in the US as purebred Holstein, purebred Jersey, and their crosses had been genotyped [7].
2. No genotype is available on a future progeny. Even if the future progeny is in the form of an embryo, embryo genotype screening is still not commercially well-established.
3. Pedigree (numerator) relationship coefficients make ties between genotyped and non-genotyped and within non-genotyped animals.
4. Pedigree structure is used in many genotype imputation procedures.

This software note introduces the free and open-source R package FnR and its functionalities. This R package answers three major questions for a pedigreed population.

1. What are the inbreeding coefficients of individuals in the population?
2. What is the inbreeding coefficient of a future progeny between a pair of individuals?
3. What are the numerator relationship coefficients between pairs of individuals?

Furthermore, it can resume calculating inbreeding coefficients for new animals added to the pedigree (or hypothetical progenies of possible mates).

## Implementation

The R package FnR (“F” stands for inbreeding coefficient, and “R” stands for relationship coefficient) consists of two functions. One for calculating the inbreeding coefficients and resuming it, and the other for the calculation of relationship coefficients between two given sets of individuals. Both functions receive a numeric pedigree data frame as input, with missing parents denoted as 0. The R package has no dependencies on other R packages and is written in base-R.

### resume_inbreed

**Fig. 1:**
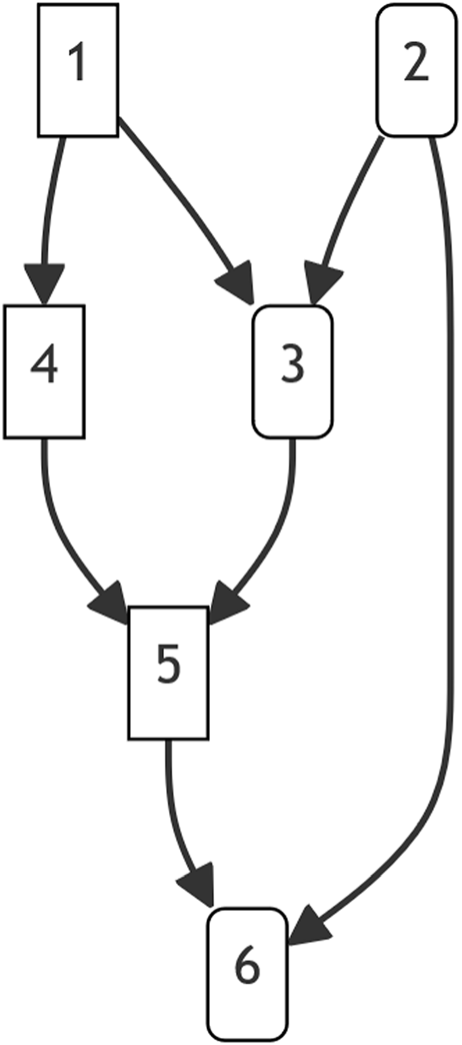
The sample pedigree of six animals. Females are shown with rounded corners

The resume_inbreed function calculates inbreeding coefficients in a population. It also allows resuming the calculation of inbreeding coefficients for new animals in the pedigree, providing the vector of previously calculated inbreeding coefficients via the argument f (optional). Parents’ inbreeding coefficients are required for the calculation of **d** coefficients, and **d** coefficients are required for the calculation of inbreeding coefficients. To speed up the resuming process, the user might provide the vector of **d** coefficients corresponding to the provided inbreeding coefficients via the argument d (optional). In that case, **d** coefficients are calculated only for animals whose inbreeding coefficient is not provided. Note that if there are any changes to the previous pedigree other than new appended animals, the inbreeding coefficients should be calculated from scratch simply by skipping arguments f and d. Alternatively, inbreeding coefficients (and **d** coefficients if available) are provided before the first change occurs in the pedigree. For example, if new animals are appended to the sample pedigree (Fig. 1) and the dam of calf 6 is changed to 3, inbreeding coefficients of animals 1–5 are provided.

The resume_inbreed function returns the vector of inbreeding coefficients for the whole population. The user might export **d** coefficients alongside the inbreeding coefficients for future use (i.e., to resume the calculation of inbreeding coefficients for new animals in the pedigree) using the argument export_d = TRUE. In that case, instead of the vector of inbreeding coefficients, a list containing the vector of inbreeding coefficients and the vector of **d** coefficients is returned.

### calcR

The calcR function generates dummy progenies between two given sets of individuals (provided to set1 and set2 arguments). The two sets should have no overlap. The argument type takes one of the three values “notdam-notsire”, “sire-sire”, or “dam-dam”, and defines what goes into set1 and set2. For example, if type = “notdam-notsire” (default) or if type is not set, set1 receives any non-dam individual(s), and set2 receives any non-sire individual(s). Similarly, if type = “sire-sire”, set1 and set2 receive only sires.

The calcR function incorporates the function resume_inbreed to calculate inbreeding coefficients for the dummy progenies. As such, if the inbreeding coefficients of the (real) population (without dummy progenies) or a part of it (i.e., previous estimates from a smaller pedigree) are available, those can be provided to speed up the procedure via the argument f (optional). The procedure can further be sped up by the availability of **d** coefficients corresponding to the provided inbreeding coefficients via the argument d (optional).

If the inbreeding and **d** coefficients are provided, those do not necessarily need to be for the whole population. Those can be from the 1^st^ to the *n*^th^ individual in the pedigree, considering that the pedigree is chronologically ordered (i.e., parents appear before progeny). The provided **d** coefficients (if any) should correspond to the provided inbreeding coefficients. resume_inbreed resumes calculating the missing inbreeding and **d** coefficients. This is helpful if the pedigree changes. Then, inbreeding coefficients (and **d** coefficients if available) are used up to the point where the first change has occurred.

## Materials

A sample pedigree of six animals from Table 2.1 of Mrode [6] was used for illustration purposes (Fig. 1). A pedigree subset of the New Zealand dairy cattle population, containing 2,721,252 animals, was used to benchmark the program (runtime and memory usage) for a large pedigree. Numerator relationship coefficients between two non-overlapping sets of 250 random non-dams and 800 random non-sires were calculated once without knowledge on the population’s inbreeding coefficients, once with knowledge on the inbreeding coefficients, and once with knowledge on both the inbreeding and **d** coefficients of the population. The analyses were performed on a t3.micro instance of Amazon Web Services with an Ubuntu 20.04 OS, which is equipped with the 1^st^ or 2^nd^ generation Intel Xeon Platinum 8000 series processor with a Turbo CPU clock speed of up to 3.1 GHz, 2 vCPUs, and 1 GB of memory (https://aws.amazon.com/ec2/instance-types/t3/). The R package FnR [8] was used throughout the study.

## Methods

The numerator relationship matrix (**A**) contains inbreeding and relationship coefficients. Calculating **A** or a partition of it has been computationally expensive and relatively slow. However, several fast and efficient methods have been developed [9–11]. There are direct and indirect methods for the calculation of **A** or a partition of it [11]. The indirect methods rely on the inbreeding coefficient estimates [10] or $\textbf{A}^{-1}$ [9, 11], since there are fast and efficient methods available for the calculation of inbreeding coefficients [9, 12–16] and $\textbf{A}^{-1}$ [12, 17, 18].

The R package FnR makes use of the method of Meuwissen and Luo [14] for the calculation of inbreeding coefficients, and the method of Van Vleck [10] for calculating a partition of **A** (i.e., relationships between pairs of individuals). The method of Van Vleck [10] is based on an innovative but a simple concept: the relationship coefficient between parents is twice the inbreeding coefficient of their progeny. As such, the progeny’s inbreeding coefficient is used to estimate the relationship coefficient between its parents. A dummy progeny is introduced to the pedigree when a pair of individuals do not have a progeny together. It is particularly important where a pair of individuals cannot have a progeny together (e.g., both are of the same sex, one is not alive or not at the breeding age).

Wright [5] devised the formula for the inbreeding coefficient: $F_X = \frac{1}{2} \sum \left[ (\frac{1}{2})^n (1 + F_A) \right]$, where $F_A$ is the inbreeding coefficient of the common ancestor *A*, and *n* is the number of path lines connecting parents (*S* and *D*) of *X*. According to Emik and Terrill [19], the relationship coefficient between parents is: $R_{SD} = \sum \left[ (\frac{1}{2})^n (1 + F_A) \right]$. Thus, $R_{SD} = 2F_X$.

The R package FnR generates a dummy progeny for any pair of individuals whose numerator relationship coefficient is to be calculated, regardless of whether they have a progeny. This is because the computational cost of calculating inbreeding coefficients is very low. Determining whether a pair of individuals have a progeny, randomly picking a progeny if they have multiple progenies, and creating a dummy progeny if no progeny was found is computationally more costly.

Given two distinct sets of individuals to calculate relationship coefficients between them, dummy progenies are introduced, and their inbreeding coefficients are calculated (which requires the calculation of inbreeding coefficients for real animals in the pedigree). The calculation of relationship coefficients between pairs of individuals using dummy progenies’ inbreeding coefficients can be done via matrix multiplications. The matrix multiplications involve $\textbf{R} = 2 \textbf{P}_1^{\prime } \text {diag}(\textbf{F}_d) \textbf{P}_2$, where **R** is an off-diagonal block of **A**, $\textbf{P}_1$ and $\textbf{P}_2$ are $mn \times m$ and $mn \times n$ parent incidence matrices (with coefficients 0 and 1), corresponding to *m* and *n* individuals in the two sets and *mn* dummy progenies, and $\textbf{F}_d$ is the vector of inbreeding coefficients for dummy progenies.

Considering the sample pedigree (Fig. 1), dummy progenies 7–10 are introduced for calculating the relationship coefficients between 1 and 4, and 3 and 6, where 7 & 8 are sired by 1, 9 & 10 are sired by 4, 7 & 9 have 3 as dam, and 8 & 10 have 6 as dam. Then, $$\textbf{P}^{\prime }_1 = \left[ \begin{array}{cccc} 1 & 1 & 0 & 0 \\ 0 & 0 & 1 & 1\end{array}\right]$$, $$\textbf{P}_2 = \left[ \begin{array}{cc} 1 & 0 \\ 0 & 1 \\ 1 & 0 \\ 0 & 1 \end{array}\right]$$, and $\textbf{F}_d = \left( 1/4, 1/8, 1/8, 5/32 \right)$. However, $2\textbf{P}_1^{\prime } \text {diag}(\textbf{F}_d) \textbf{P}_2$ multiplications were avoided in R package FnR, by directly forming **R**, and copying twice the inbreeding coefficients of dummy progenies in the designated cells of **R** at once (no loop involved).

## Results and discussions

The runtime and peak memory usage for calculating the numerator relationship coefficients between a sample of 250 random non-dams and 800 random non-sires from a pedigree of 2,721,252 animals are reported in Table 1. The results showed a considerable reduction in the runtime when the inbreeding coefficients are available, and there is no need to calculate them (except for the 200,000 dummy progeny). The availability of **d** coefficients further reduced the runtime slightly. In this example, inbreeding and **d** coefficients were provided for the whole (real) population. However, it does not need to be like this. Inbreeding and **d** coefficients can be provided (optional) from the previous calculation of inbreeding coefficients with less number of animals in the pedigree. If there are changes to the previous pedigree, the inbreeding and **d** coefficients should be supplied before the occurrence of the first change.

**Table 1:** Runtime (mm:ss) and the (session’s) peak memory usage (MB) estimates for calculating numerator relationship coefficients between two distinct random sets of 250 non-dams and 800 non-sires, from a pedigree of 2,721,252 animals, and different combinations of the availability of inbreeding and **d** coefficients (**d** = diag(**D**); **D** is a diagonal matrix in $\textbf{A} = \textbf{TDT}'$). Inbreeding and **d** coefficients were provided for the whole pedigree (without dummy progeny). The analyses were run on a t3.micro instance of Amazon Web Services

Available coeff.	Runtime	Peak MB^a^
NA	3:45	660.7
Inbreeding	1:08	698.3
Inbreeding and **d**	0:54	741.2

^a^It took 99 MB of memory to load R

When inbreeding coefficients are not provided, those are calculated for the whole population and dummy progenies. When inbreeding coefficients are provided, first, **d** coefficients are calculated for the animals whose inbreeding coefficients are provided. Then, inbreeding coefficients are calculated for the rest of the real animals (if inbreeding coefficients are not provided for the whole population) and dummy progenies. When inbreeding and **d** coefficients are provided, inbreeding coefficients will be calculated for the rest of the real animals and dummy progenies.

The pedigree data frame, the vector of inbreeding coefficients, and the vector of **d** coefficients took 31.1, 20.8, and 20.8 MB of memory, respectively. The two sets of (250 and 800) animals took 1,048 and 3,248 bytes of memory. It also took 99 MB of memory to load R.

Calculating inbreeding coefficients is fast enough to discourage unnecessary data handling costs. However, suppose the population is very large, and the set of animals whose inbreeding coefficients are of interest or the two sets of animals whose relationship coefficients are of interest are small. In that case, one may consider extracting a pedigree subset by tracing the pedigree upward from the animals of interest.

As an active project, this R package may undergo further development. Currently, no specific development is planned. However, possible developments might include implementing other methods for calculating inbreeding and numerator relationship coefficients.

## Conclusion

The R package FnR is a free and open-source tool for calculating inbreeding and numerator relationship coefficients between two sets of individuals in a pedigreed population. It can help conservation and breeding programs monitor the inbreeding rate in the population, find the numerator relationship coefficients between pairs of individuals, and the inbreeding rate of their prospective progeny (half of their relationship coefficient). The program showed to be time and memory efficient over a relatively large pedigree of 2,721,252 animals and 200,000 dummy progenies, on a low-end computer with 2 vCPUs and 1 GB of memory. The program provides the possibility of exporting the estimated inbreeding and **d** coefficients for future use (i.e., new animals appended to the pedigree). Though using the previously calculated inbreeding coefficients considerably reduced the computational time, the additional gain by using the **d** coefficients was marginal, and it might be unnecessary.

## Data Availability

The large pedigree data is proprietary of Livestock Improvement Corporation (Hamilton, New Zealand) and cannot be shared.

## References

[1] Charlesworth D, Willis JH (2009). The genetics of inbreeding depression. Nat Rev Genet..

[2] Leroy G, Mary-Huard T, Verrier E, Danvy S, Charvolin E, Danchin-Burge C. Methods to estimate effective population size using pedigree data: Examples in dog, sheep, cattle and horse. Genet Sel Evol. 2013;45(1). 10.1186/1297-9686-45-1. 10.1186/1297-9686-45-1 PMC3599586 23281913

[3] Notter DR (1999). The importance of genetic diversity in livestock populations of the future. J Anim Sci..

[4] Falconer DS, Mackay TFC (1996). Introduction to quantitative genetics.

[5] Wright S (1922). Coefficients of inbreeding and relationship. Am Nat..

[6] Mrode RA (2005). Linear models for the prediction of animal breeding values.

[7] Cesarani A, Lourenco D, Bermann M, Nicolazzi EL, VanRaden PM, Misztal I (2024). Single-step genomic predictions for crossbred Holstein and Jersey cattle in the United States. JDS Comms..

[8] Nilforooshan MA. FnR: Inbreeding and numerator relationship coefficients. 2024. https://CRAN.R-project.org/package=FnR. Accessed 12 July 2024. 10.1186/s12862-024-02285-4 PMC11256478 39026190

[9] Colleau JJ. An indirect approach to the extensive calculation of relationship coefficients. Genet Sel Evol. 2002;34(409). 10.1186/1297-9686-34-4-409. 10.1186/1297-9686-34-4-409 PMC2705453 12270102

[10] Van Vleck LD (2007). Computing numerator relationships between any pair of animals. Genet Mol Res..

[11] Nilforooshan MA, Garrick D, Harris B. Alternative ways of computing the numerator relationship matrix. Front Genet. 2021;12. 10.3389/fgene.2021.655638. 10.3389/fgene.2021.655638 PMC8356081 34394180

[12] Quaas RL (1976). Computing the diagonal elements and inverse of a large numerator relationship matrix. Biometrics..

[13] Tier B (1990). Computing inbreeding coefficients quickly. Genet Sel Evol..

[14] Meuwissen THE, Luo Z. Computing inbreeding coefficients in large populations. Genet Sel Evol. 1992;24(305). 10.1186/1297-9686-24-4-305.

[15] Sargolzaei M, Iwaisaki H (2005). Comparison of four direct algorithms for computing inbreeding coefficients. Anim Sci J..

[16] Sargolzaei M, Iwaisaki H, Colleau JJ (2005). A fast algorithm for computing inbreeding coefficients in large populations. J Anim Breed Genet..

[17] Henderson CR (1976). A simple method for computing the inverse of a numerator relationship matrix used in prediction of breeding values. Biometrics..

[18] Nilforooshan MA (2022). A new computational approach to Henderson’s method of computing the inverse of a numerator relationship matrix. Livest Sci..

[19] Emik LO, Terrill CE (1949). Systematic procedures for calculating inbreeding coefficients. J Hered..

